# Stability of Zika Virus Antibodies in Specimens from a Retrospective Serological Study

**DOI:** 10.4269/ajtmh.21-0564a

**Published:** 2021-07-26

**Authors:** Ning Zhang, Ruiyan Zhang, Teng Chen, Xiaocheng Li, Shubo Zhang, Wenchen Han, Bin He, Hang Yin, Ying Meng, Jinghui Zhao

**Affiliations:** Institute of Biopharmaceutical Research Liaocheng University Liaocheng 252059, China E-mails: zhangning1111@126.com and zry147896@163.com; Key Laboratory of Jilin Province Zoonoses Prevention and Control Laboratory of Epidemiology Institute of Military Veterinary Medicine Academy of Military Medical Sciences Changchun 130122, China E-mail: 1093303757@qq.com; Jilin Dabeinong Agriculture and Animal Husbandry Technology Co., Ltd. Changchun 130102, China Beijing Dabeinong Technology Group Co., Ltd. Beijing 100080, China E-mails: zhaojinghui8791@hotmail.com, lxch_215@yeah.net, 412648487@qq.com, wlyweiliyan@sina.com, vet126@163.com, yinhang510@163.com, 1094101240@qq.com, and zhaojinghui8791@hotmail.com Published online July 26, 2021.

Dear Sir,

In May 2021, Sasmono et al.^1^ published their interesting work, which reported on widescale use of antibody seroprevalence to examine the history of Zika virus (ZIKV) transmission in Indonesia. The specimens for this retrospective seroprevalence study were collected in 2014, so the stability of anti-ZIKV antibodies in the serum samples, which were stored for 6 years, should be further considered.

First, the storage conditions of the 870 serum samples, which were collected from different regions in Indonesia in 2014, were not presented in the manuscript. According to the authors, “A total of 870 serum samples from healthy 5- to 9-year-old children, which had been collected from 30 districts in 14 provinces during October and November 2014, were tested.” The storage conditions of the long-term stored sera samples should have been included in the article, and this information will be helpful in interpreting the data.

Second, there has been no report that anti-ZIKV antibodies are stable during long-term storage at –20°C or –80°C. The stability of antibody titers against ZIKV may influence the results and conclusions of seroprevalence studies. It is worth noting that the relationship between anti-ZIKV antibody titers and storage conditions should be investigated further.
